# Novel CTC Detection Method in Patients with Pancreatic Cancer Using High-Resolution Image Scanning

**DOI:** 10.3390/cancers17223640

**Published:** 2025-11-13

**Authors:** Takahiro Manabe, Tomoyuki Okumura, Kenji Terabayashi, Takahisa Akashi, Teo Yi Rui, Yoshihisa Numata, Naoya Takeda, Akane Yamada, Nana Kimura, Mina Fukasawa, Tatsuhiro Araki, Kosuke Mori, Yusuke Kishi, Kisuke Tanaka, Tomohiro Minagawa, Takeshi Miwa, Toru Watanabe, Katsuhisa Hirano, Shinichi Sekine, Isaya Hashimoto, Kazuto Shibuya, Isaku Yoshioka, Koshi Matsui, Tohru Sasaki, Tsutomu Fujii

**Affiliations:** 1 Department of Surgery and Science, Faculty of Medicine, Academic Assembly, University of Toyama, Toyama 9300194, Japan; tmanabe@med.u-toyama.ac.jp (T.M.);; 2 Department of Mechanical and Intellectual Systems Engineering, Faculty of Engineering, University of Toyama, Toyama 9300194, Japan

**Keywords:** high-resolution image scanning, circulating tumor cells, negative enrichment, pancreatic ductal adenocarcinoma, liquid biopsy

## Abstract

Background/Objectives: Appropriate biomarkers are necessary for early diagnosis and multidisciplinary treatment of pancreatic ductal adenocarcinoma (PDAC). In recent years, the clinical utility of circulating tumor cells (CTC) as biomarkers for various can-cers has been reported; however, their detection rate in PDAC remains low, and clinical evidence is not yet established. CTC detection methods with high reliability and per-formance are essential for clarifying the importance of CTC in patients with PDAC. Methods: A total of 5 mL peripheral blood samples were collected from 38 patients newly diagnosed with PDAC and 17 healthy controls. Negatively enriched cells were immunofluorescently stained with EpCAM-phycoerythrin and cell surface vi-mentin-fluorescein isothiocyanate (CSV). Images were automatically captured using an all-in-one fluorescence microscope. Cellular regions were detected from these images, and the average luminance of the cellular regions was calculated. A total of 9086 and 1071 cell images were obtained from patients with PDAC and healthy controls, respec-tively. Results: In the EpCAM assay, a threshold that included 95% of healthy individuals was optimal for distinguishing patients with PDAC from healthy controls, with a sensi-tivity, specificity, and area under the curve of 0.74, 0.76, and 0.84, respectively. At this threshold, the CTC-positivity rate in patients with PDAC was 76.3%. Conversely, the CSV assay failed to demonstrate a valid threshold to distinguish patients with PDAC from healthy controls. No significant differences were found between CTC and clini-copathological features among patients with PDAC. Conclusions: The method using high-resolution image scanning has the potential to identify CTC with greater objectiv-ity by quantifying cell luminance values.

## Simple Summary

Pancreatic ductal adenocarcinoma is one of the most lethal cancers, and its early diagnosis remains challenging. Circulating tumor cells, which are found in the bloodstream, have emerged as a promising marker for cancer diagnosis and treatment monitoring, but their detection rate in pancreatic ductal adenocarcinoma has been low. In this study, we developed a new detection method that combines negative enrichment with high-resolution image scanning. This approach allows an objective measurement of antigen expression and improves the accuracy of detecting circulating tumor cells. Our findings suggest that this method may provide a reliable and minimally invasive biomarker for the diagnosis of pancreatic ductal adenocarcinoma and has the potential to support earlier detection and better treatment strategies in the future.

## 1. Introduction

Pancreatic ductal adenocarcinoma (PDAC) is the fourth leading cause of cancer-related death, with a dismal prognosis and an approximate 5-year survival rate of 9%. Owing to the absence of early symptoms and effective screening, PDAC is often diagnosed at an advanced stage [1]. However, in the last decade, novel chemotherapeutic regimens, such as FOLFIRINOX and gemcitabine along with nab-paclitaxel (GnP), have incrementally improved the prognosis in patients with PDAC. In addition, multimodal treatment combining induction chemotherapy, surgery, and subsequent chemoradiotherapy has begun to show promising results [2]. Appropriate biomarkers are essential for both early diagnosis and guiding multimodality treatment in PDAC. Frequently used biomarkers of PDAC include carcinoembryonic antigen, carbohydrate antigen 19-9 (CA19-9), pancreatic cancer-associated antigen Duke-PAN-2, and S-pancreas-1 antigen. Among these, CA19-9 is particularly valuable but has limitations in early diagnosis and in cases of obstructive jaundice, highlighting the need for more sensitive biomarkers [3].

Recently, circulating tumor cells (CTC) have attracted attention as biomarkers for various cancers; CTC are a rare subset of disseminated tumor cells in the bloodstream of patients with solid tumors, shed from primary tumors and considered pivotal for distant metastasis, with important oncological implications. As a form of liquid biopsy, CTC has shown utility in various areas, including diagnosis, disease progression assessment, and therapeutic efficacy evaluation [4]. However, evidence from CTC studies in patients with PDAC remains lacking. Using CellSearch (Menarini Biosystems, Bologna, Italy), the only FDA-approved and currently gold standard method for CTC detection, the detection rate of CTC in patients with PDAC is low, ranging from 7 to 48%, which is the lowest among solid tumors [5,6]. To clarify the importance of CTC in patients with PDAC, establishing a reliable and high-performance method for CTC detection is essential.

The workflow of CTC research is divided into two stages, enrichment and identification. Selecting an appropriate method based on the tumor characteristics and the study objectives proves important. CTC enrichment methods using immunomagnetic separation are broadly classified into positive and negative enrichment methods. Positive enrichment provides high purity but lower recovery rates, while negative enrichment offers higher recovery rates but with reduced purity. The inverse relationship between purity and recovery is a critical factor in selecting an appropriate enrichment method. For cancers such as PDAC, negative enrichment may be effective for enhancing CTC recovery. However, even with improved recovery rates, identifying CTC from low-purity populations proves difficult, which remains a major challenge for downstream analysis. After enrichment, only a small fraction of the hundreds to thousands of cells are likely CTC, making accurate identification challenging even for an experienced pathologist, which can be time-consuming, while the accuracy of identification cannot be guaranteed [7]. Negative enrichment requires efficient and objective CTC identification for downstream analysis.

High-resolution image scanning offers excellent objectivity and is promising for this purpose, particularly in combination with recent advances in artificial intelligence, which may further improve CTC separation efficiency, reduce costs, and enhance detection sensitivity and accuracy [8,9,10]. However, to date, no studies have applied high-resolution image scanning for CTC analysis in patients with PDAC, and standardized criteria and methodology for CTC identification remain unclear, necessitating further basic investigation. In this study, we aimed to evaluate the diagnostic utility of CTC detection in PDAC by quantifying antigenic marker expression in negatively enriched cells using high-resolution image scanning. This study was designed to assess diagnostic performance only; prognostic implications were not evaluated, as survival and outcome data were not included in the present dataset.

## 2. Materials and Methods

### 2.1. Patient Recruitment and Sample Collections

This study was approved by the Ethical Review Board of Toyama University Hospital (R2021061) and conducted in accordance with the Declaration of Helsinki. Written informed consent was obtained from all the participants. Peripheral blood samples were collected from 38 patients newly diagnosed with PDAC and 17 healthy controls. Patient samples were collected between January 2023 and December 2023. The eligibility criteria for patients were (i) pathologically confirmed by endoscopic ultrasound-fine needle aspiration, (ii) no PDAC treatment prior to enrollment, and (iii) no history of synchronous double cancer. The healthy controls had no history of malignant disease. All patients with PDAC were staged using abdominal enhanced computed tomography, enhanced magnetic resonance imaging, and staging laparoscopy including peritoneal washing cytology (CY). The tumor-node-metastasis staging system for pancreatic tumors in the 8th edition of the Union for International Cancer Control (UICC) was applied [11] (Supplementary Table S1).

### 2.2. Sample Collection and CTC Enrichment

**Figure 1:**
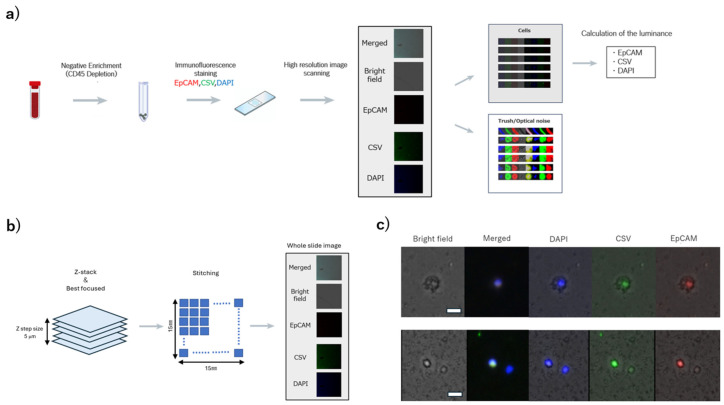
Schematic of overall design. (**a**) Flowchart of experimental procedures. (**b**) Image capture and pre-processing. Images are automatically captured with an all-in-one fluorescence microscope (BZ-X800) using a objective with a Z step size of 5 μm. (**c**) Cut-out images of CTC candidate cells (scale bar: 20 μm). Red fluorescence indicates EpCAM staining, green fluorescence indicates cell-surface vimentin (CSV), and blue fluorescence indicates nuclear staining with DAPI. CTC, circulating tumor cells.

Peripheral blood samples (5 mL) were collected prior to any surgical intervention or systemic chemotherapy and drawn into ethylenediaminetetraacetic acid (EDTA) tubes. Samples were processed within 4 h of collection. Peripheral blood was subjected to CD45 depletion using the RosetteSep™ kit (StemCell Technologies, Vancouver, BC, Canada) according to the manufacturer’s instructions. After RBC lysis using BD Pharm Lyse™ lysing solution (Becton, Dickinson and Company, Franklin Lakes, NJ, USA) and fixation with 4% paraformaldehyde, enriched cells were stained using manual immunofluorescence with EpCAM-phycoerythrin (clone REA764; MACS Miltenyi Biotec, Cologne, Germany) and cell surface vimentin-fluorescein isothiocyanate(CSV) (clone 84-1; ABNOVA, Taipei, Taiwan). Nuclei were stained with 4′, 1, 6′-diamidino-2-phenylindole (DAPI) (Thermo Fisher, Waltham, MA, USA). The stained cells were transferred to microscope slides and prepared for imaging (Figure 1a).

### 2.3. High-Resolution Image Scanning and Image Processing

Images were automatically captured using an all-in-one fluorescence microscope (BZ-X800, KEYENCE, Osaka, Japan) through a Plan Apochromat 20x objective (NA0.75, BZ-PA20, KEYENCE, Osaka, Japan) with a Z-step size of 5 μm. Red fluorescence was detected with a TRITC filter (ex: 545/25 nm, em: 605/70 nm, dichroic: 565 nm; OP-87764, KEYENCE, Osaka, Japan) at 1/20 s exposure time. Green fluorescence was detected with a GFP filter (ex: 470/40 nm, em: 525/50 nm, dichroic: 495 nm, OP-87763; KEYENCE, Osaka, Japan) at 1/15 s exposure time. Blue fluorescence was detected using a DAPI-V filter (ex: 395/25 nm, em: 460/50 nm, dichroic: 425 nm, OP-88359; KEYENCE, Osaka, Japan) with an exposure time of 1/250 s. The image-stacking function of the BZ-X Analyzer software (version 1.4.1; KEYENCE, Osaka, Japan) was used to stitch the captured images (Figure 1b). The captured images were subsequently processed using an algorithm developed in cooperation with the Department of Mechanical and Intellectual Systems Engineering, Faculty of Engineering, University of Toyama [8]. This process consists of two steps: (1) detection of cellular regions and (2) calculation of the average luminance of the cellular regions (Figure 1c).

#### 2.3.1. Detection of Cellular Regions

DAPI-expressing regions in the captured image were converted to grayscale, and the foreground regions were extracted using binarization. After removing the noise by a closing process that performs three expansions and three contractions, the cell regions were extracted using the watershed transformation. Excluding non-cellular images (debris and optical noise) from the detected cellular regions is necessary. A proprietary machine-learning algorithm trained on an annotation library containing approximately 10,000 cell and non-cellular images assisted in the sorting of cell and non-cellular (e.g., debris, optical noise) regions. The selected images were submitted to trained reviewers, and only cell images were used for luminance analysis. (Supplementary Method S1, Supplementary Table S2).

#### 2.3.2. Calculation of the Average Luminance of Cellular Regions

To minimize the effects of differences in staining conditions and nonspecific luminescence in each image, the background average luminance uniformity was performed for each cell image. The EpCAM and CSV images of the extracted cell regions were grayscale and binarized, respectively. The foreground regions were extracted, the luminance of each image was measured, and the luminance average was calculated.

### 2.4. Threshold Settings

A dataset of 10 randomly selected healthy controls was used to set the thresholds. EpCAM and CSV luminance were measured for the detected cells, and the thresholds were set to include 99, 95, and 90% of all the cells for both EpCAM and CSV.

### 2.5. Statistical Analysis

Correlations between CTC counts and clinicopathological data were analyzed using Fisher exact tests or chi-square, and Mann–Whitney U test. Receiver Operating Characteristic (ROC) analysis was performed to evaluate areas under the curve (AUC), sensitivity (SN), specificity (SP), positive predictive values, and negative predictive values. The optimal cutoff value was determined according to the maximum value of the Youden J index. All statistical analyses were performed using JMP Pro ver.17 (SAS Institute, Cary, NC, USA). A two-sided *p*-value < 0.05 was considered significant.

## 3. Results

### 3.1. Acquisition of Cell Images and Measurement of Luminance

We obtained 9086 and 1071 cell images from 38 patients with PDAC and healthy controls, respectively. Of these, luminance was measured for both EpCAM and CSV (Supplementary Figure S1). In the EpCAM assay, the mean luminance was 16.1 (4.2–227.3) for the patient with PDAC and 16.3 (2.7–197.1) lumens for the healthy control groups, without significant difference (*p* = 0.689). In the CSV assay, the average was 29.4 (6.6–237.0) and 34.4 (8.4–221.0) lumens in the patient with PDAC and healthy control groups, respectively, showing a significant difference (*p* < 0.0001).

### 3.2. Setting Thresholds and Counting CTC Candidate Cells at Each Threshold

**Figure 2:**
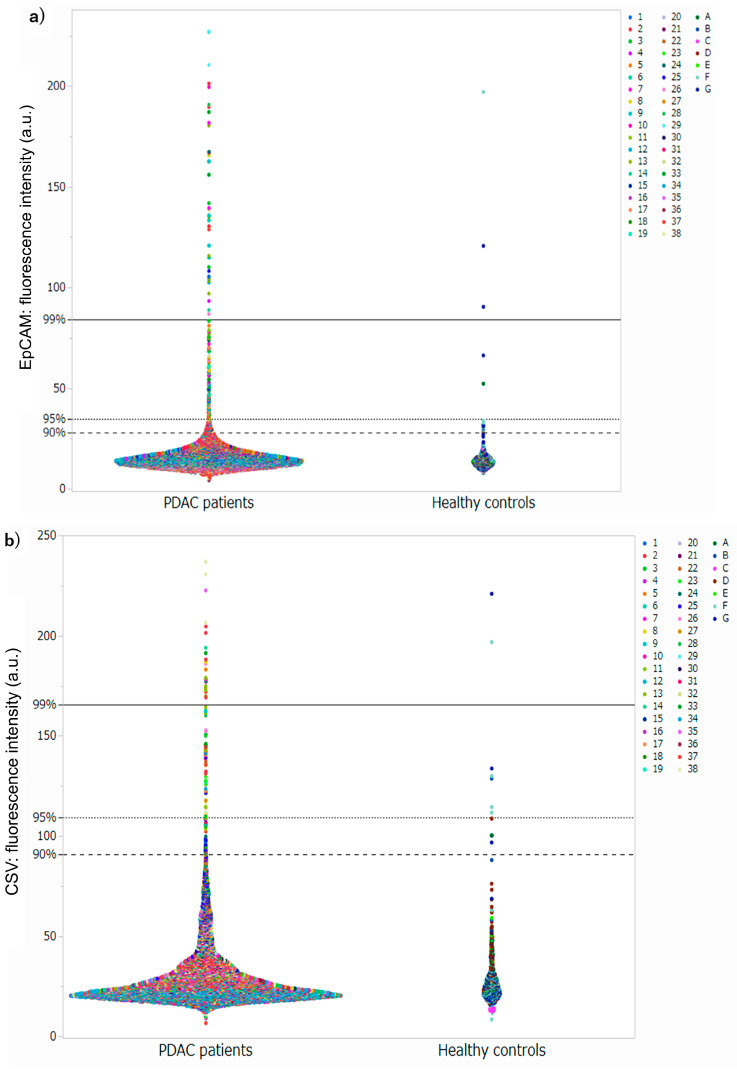
Distribution of luminance values in the test set. (**a**) Luminance values in EpCAM assay (1~38: patients with PDAC, A~G: Healthy controls). (**b**) Luminance values in CSV assay (1~38: patients with PDAC, A~G: Healthy controls). CSV, cell surface vimentin; PDAC, pancreatic ductal adenocarcinoma.

**Figure 3:**
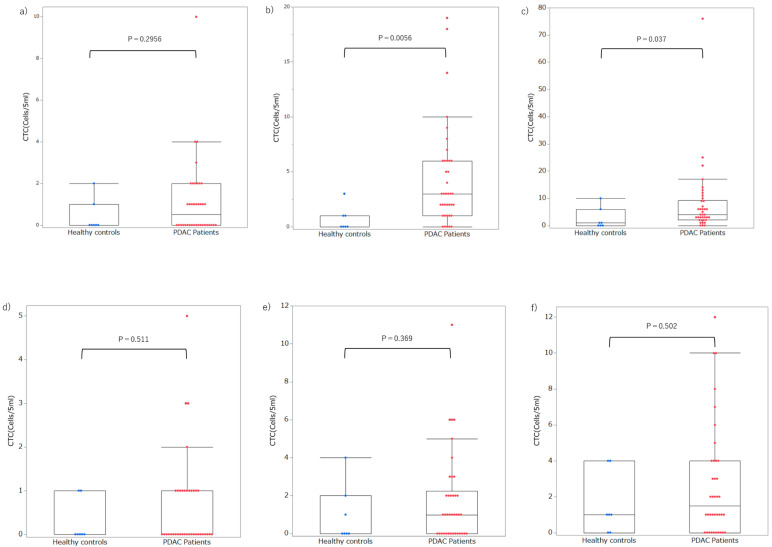
Number of candidate CTC (**a**) Number of candidate CTC in EpCAM (99). (**b**) Number of candidate CTC in EpCAM (95). (**c**) Number of candidate CTC in EpCAM (90). (**d**) Number of candidate CTC in CSV (99). (**e**) Number of candidate CTC in CSV (95). (**f**) Number of candidate CTC in CSV (90). CTC, circulating tumor cells; CSV, cell surface vimentin. Colored dots represent individual data points from each sample (blue: healthy controls; red: patients with PDAC). Statistical significance was evaluated using the Mann–Whitney U test. Data are presented as median (interquartile range). The box represents the interquartile range, the horizontal line indicates the median, the whiskers denote the minimum and maximum values within 1.5 × IQR, and outliers are shown as individual points.

The luminance threshold was determined using 336 images from 10 randomly selected healthy controls. For EpCAM, the luminance threshold to include 99% of the cells in the healthy controls was 84.3 lumens, 34.5 lumens for 95% of the cells, and 28.0 lumens for 90% of the cells. For CSV, the luminance thresholds to include 99%, 95%, and 90% of the cells were 165.4, 109.4, and 91.0 lumens, respectively. Hereafter, the respective luminance thresholds are referred to as EpCAM (99) and CSV (95). Cells exceeding each threshold were counted from 38 patients with PDAC and seven healthy controls in the test set (Supplementary Table S3 and Figure 2). For the EpCAM assay, the detection rates in patients with PDAC were 50.0%, 86.8%, and 92.1% for the EpCAM (99), EpCAM (95), and EpCAM (90) thresholds, respectively, and the median number of cells detected was 0.5 (0–10), 3 (0–19), and 4.5 (0–79). In contrast, the corresponding detection rates in healthy controls were 28.6%, 42.9%, and 57.1%, respectively, with a median number of cells detected of 0 (0–2), 0 (0–3), and 1 (0–10). A significant difference was observed in the number of cells detected between patients with PDAC and healthy controls using the thresholds of EpCAM (95) (*p* = 0.0056) and EpCAM (90) (*p* = 0.037) (Figure 3a–c). In the CSV assay, the detection rates in patients with PDAC were 42.1%, 63.2%, and 73.7% for thresholds of CSV (99), CSV (95), and CSV (90), respectively, and the median number of cells detected was 0 (0–6), 1 (0–12), and 1 (0–12). In contrast, the detection rates in healthy controls were 28.6%, 57.1%, and 71.4%, and the median number of cells detected was 0 (0–1), 1 (0–4), and 1 (0–4), respectively (Figure 3f). No significant differences were observed in the number of cells detected between patients with PDAC and healthy controls at any threshold.

### 3.3. Setting Optimal Thresholds and Distributions of CTC in Patients with PDAC

**Figure 4:**
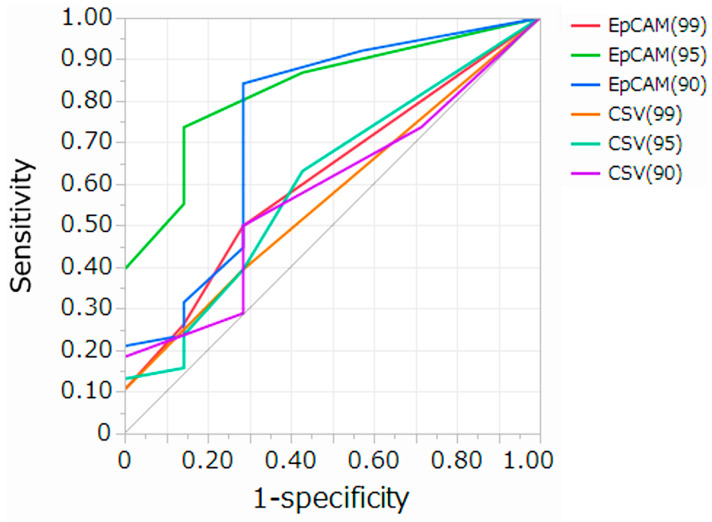
Receiver operating characteristic (ROC) curves comparing the diagnostic performance of EpCAM and CSV assays for distinguishing pancreatic ductal adenocarcinoma (PDAC) patients from healthy controls.

**Figure 5:**
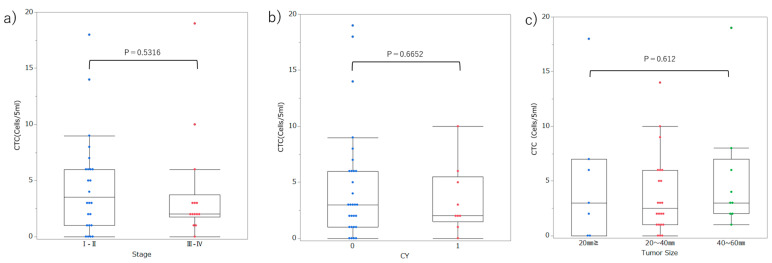
Association between the number of CTC and clinicopathological data in patients with PDAC. (**a**) Early stage (stage I–II) and advanced stage (stage III–IV). (**b**) CY positivity. (**c**) Tumor size. Statistical differences were assessed using the Mann–Whitney U test or Fisher’s exact test, as appropriate. Data are presented as median (interquartile range). The box represents the interquartile range, the horizontal line indicates the median, the whiskers denote the minimum and maximum values within 1.5 × IQR, and outliers are shown as individual points.

At each threshold, ROC curves and AUC analyses were performed to assess the diagnostic ability to distinguish patients with PDAC from healthy controls. Moreover, we compared the AUC for each threshold value using the Delong test (Figure 4 and Table 1). EpCAM (95) was the most suitable threshold to distinguish patients with PDAC from healthy controls, with an SN of 0.74 and SP of 0.76, using a cutoff value of 2 cells/5 mL, according to the Youden index. The comparison of the AUCs showed that the performance of EpCAM (95) was significantly superior to that of EpCAM (99), CSV (99), CSV (95), and CSV (90) (AUC EpCAM (95) = 0.84, Delong test *p-value* vs. *EpCAM* (99) = 0.0002, Delong test *p-value* vs. *CSV* (99) = 0.0002, Delong test *p-value* vs. *CSV* (95) = 0.0201, Delong test *p-value* vs. *CSV* (90) = 0.0021, respectively). We defined CTC as cells with luminance higher than that of the threshold EpCAM (95) and investigated the association between CTC and clinicopathological data in patients with PDAC. The number of CTC showed no significant differences between early (stage I–II) and advanced (stage III–IV) patients, regardless of CY-positivity or tumor size (Figure 5). The positivity rate was 76.3% (29/38) using a cutoff value of 2 cells/5 mL. The positive rates of CTC did not correlate with age, sex, tumor location, TNM stage, CY, or tumor size (Table 2).

## 4. Discussion

In this study, we analyzed CTC in patients with PDAC using a combination of negative enrichment and high-resolution image scanning. High-resolution image scanning offers an objective approach to assessing CTC by quantifying cell luminance values. This method has the potential to detect not only CTC with high EpCAM expression, as captured by conventional techniques, but also those with low EpCAM expression.

Using high-resolution image scanning, we attempted to estimate the luminance range in which CTC were present by quantifying the luminance values of the cells and comparing them to those of healthy controls. EpCAM is expressed in most epithelial malignancies and 94% of PDAC tissues. However, EpCAM expression varies across PDAC with only approximately 50% of them showing high EpCAM expression [12]. Variation in EpCAM expression is expected not only in PDAC tissues but also in CTC. Indeed, a large variation in EpCAM expression has been reported in CTC isolated from patients with prostate cancer, with differences observed both between patients and within individual patients [13]. CellSearch may limit CTC detection rate in patients with PDAC due to its inability to detect CTC lacking EpCAM expression as well as those with low EpCAM expression [14]. The detection rate of CTC in patients with PDAC using CellSearch is low, ranging from 7 to 48% [5]. Consequently, research on CTC in this population has progressed more slowly than in other cancer types, such as breast, prostate, and colorectal cancers. The challenge in CTC research is to detect CTC across a broad range of variations, including those with low EpCAM expression, and identify new surface antigens as alternatives to EpCAM.

A quantitative evaluation of surface antigens would be beneficial for detecting CTC with varying expression levels; however, few studies have quantitatively measured these surface antigens [13,15], and reports on high-resolution image scanning are lacking. This imaging technique allows for the quantitative evaluation of surface antigen marker expression levels by mapping and averaging cell luminance. Background adjustments were implemented to correct the differences associated with the staining technique, followed by the calculation of luminance values. After quantifying the luminance values of the cell surface antigens, estimating the luminance range indicative of CTC presence is necessary. Three thresholds for each antibody were established using the dataset from healthy control patients, and the luminance range in which CTC were present was estimated by comparing the test sets of patients with PDAC and healthy controls. Using EpCAM (99) as the luminance threshold, the detection rate was approximately 50%, which is nearly identical to that of conventional methods, such as CellSearch, which can only detect CTC with high EpCAM expression. The luminance thresholds for EpCAM (95) and EpCAM (90) were estimated as the lower limit of the presence of EpCAM-positive CTC, revealing that EpCAM-positive CTC varies from high to low expression, even in patients with PDAC.

We also investigated CSV as an alternative CTC marker for EpCAM. CSV as an epithelial–mesenchymal transition marker has been reported to be useful in identifying mesenchymal CTC in PDAC [16]. Some studies report that CSV outperforms EpCAM in detection rates for PDAC [17], while others indicate that CSV is less effective than EpCAM and that no CTC shows only mesenchymal surface antigen [18,19]. A common challenge in CTC studies is the difficulty in comparing results owing to variations in detection and identification methods and differences in patient backgrounds, such as tumor stage. Therefore, it remains controversial whether CSV are more effective than EpCAM or merely serve as complementary markers for the diagnosis of PDAC. The prognostic significance of mesenchymal CTC also remains inconsistent [20]; while some studies have suggested that mesenchymal CTC are associated with poor prognosis [21], others have found no such correlation [22]. In our study, we were unable to establish a reliable CSV threshold to differentiate patients with PDAC from healthy controls. This could be attributed to the possibility that the number of CSV-positive CTC in patients with PDAC might have been lower than we initially expected, to a narrow luminance range for CSV-positive CTC, or to a high false-positive rate in healthy controls. Increasing the number of cellular images from patients with PDAC and healthy controls might help improve detection accuracy. However, the clinical significance of both epithelial and mesenchymal CTC is intriguing and warrants further investigation.

In this study, the detection rate in patients with PDAC was 76.8% (cutoff: ≥2 CTCs), and the number of CTC proved useful in differentiating patients with PDAC from healthy controls (AUC = 0.84; SN = 0.74; SP = 0.76, using a cutoff of 2 cells/5 mL). Previous studies examining the diagnostic performance of CTCs reported AUCs ranging from 0.83 to 0.85, similar to the values observed in this study [23,24,25]. On the other hand, although the cutoff was set at the 95% confidence level based on healthy controls, approximately 20% of them were classified as CTC-positive. Such false-positive cases are frequently observed in CTC research; in the present study, they may be attributed to non-specific fluorescence signals, such as autofluorescence or antibody cross-reactivity, or to contamination by epithelial cells during blood collection. In addition, the limited sample size of this study may have influenced the reliability of the threshold setting, and addressing these factors remains an important issue for future research.

CTC and CA19-9 have been reported to play complementary roles in the diagnosis of PDAC [24]. In our cohort of 38 patients with PDAC, 10 patients (26.3%) were CTC-positive (≥2 cells/5 mL) despite having normal CA19-9 levels. Conversely, 7 patients (18.4%) were CTC-negative while exhibiting elevated CA19-9 levels. These findings suggest that CTC detection may serve as a useful adjunctive diagnostic indicator in patients with CA19-9–negative PDAC. An important factor that improves the prognosis of PDAC is early diagnosis. CTC were detected in 17 out of 24 patients (70.8%) with early-stage PDAC using a cutoff value of ≥2 cells per 5 mL, whereas CA19-9 elevation (≥37 U/mL) was observed in 12 of 24 patients (50.0%). Although the number of cases is limited, these findings suggest that CTC detection may be useful for early PDAC diagnosis and that a complementary use of CTC and CA19-9 could potentially enhance diagnostic sensitivity. As current PDAC diagnosis relies mainly on imaging and advanced endoscopic techniques, establishing a minimally invasive liquid biopsy such as CTC analysis may contribute to earlier detection and improved patient outcomes. To our knowledge, no studies have reported the usefulness of CTC in the early detection of PDAC; future large-scale studies are needed to validate their potential.

We did not find any association between the clinicopathological background and CTC. Notably, staging laparoscopy and washing cytology were performed in all patients with PDAC without overt distant metastases, revealing no association between CY-positivity and CTC or other distant metastases [26]. The association between the clinicopathological background and CTC in patients with PDAC remains unclear. Court et al. reported an association between the number of CTCs and PDAC Stage [27], while Bissolati et al. and Okubo et al. reported higher CTC positivity in cases with distant metastases [28,29]. However, many other studies have failed to show an association between clinicopathological background and CTC. In general, CTC positivity, similar to other malignancies, may be associated with an unfavorable prognosis in patients with PDAC [30]. Variations in CTC detection methods and rates across studies complicate comparative assessments. Future studies will benefit from a standardized, efficient detection method capable of differentiating CTC by subset, such as epithelial and mesenchymal.

Immunological staining offers a straightforward and suitable approach for evaluating CTC counts compared to mRNA or DNA mutation analysis and is most appropriate when evaluating the number of CTC. While more reliable than other methods, this approach is time-consuming and yields inconsistent evaluations even among experts [7]. To enhance objectivity and efficiency, we employed high-resolution image scanning with conventional machine learning for CTC analysis. Although the method offers greater objectivity in CTC identification, it relies on manual input and is not yet a fully comprehensive analytical method. Deep learning (DL), which has advanced considerably in recent years, aligns well with image analysis and promises to enable comprehensive automated CTC analysis in the future. Studies using cell-line models have shown that DL is markedly more efficient than manual identification [8], and that it also provides higher accuracy than conventional machine learning approaches [10]. Thus, although the potential of applying DL for CTC identification in the future is beyond debate, a considerable gap still exists between analyses based on cell lines and those involving actual patient blood samples. Unlike cell lines, which provide ample reliable, and well-annotated material are typically scarce and lack detailed characterization. In addition, inter-observer variability in CTC annotation can complicate the immediate implementation of DL into CTC analysis. Overcoming these challenges will usher CTC research into a new era.

This study has several limitations. First, it was conducted at a single institution with a limited sample size; therefore, the findings should be interpreted as preliminary. Second, the molecular characteristics of CTC were not evaluated in this study. Third, multivariate logistic regression analyses were not performed because the number of CTC-positive cases was limited, and the inclusion of multiple clinical variables would not have yielded a statistically reliable model. Moreover, the primary aim of this study was to assess the diagnostic performance and feasibility of the image-based CTC detection system, rather than to develop a predictive clinical model; therefore, univariate analyses were considered appropriate for the study scope. Future studies with larger, multicenter patient cohorts are warranted to validate the diagnostic utility of this CTC detection system and to further investigate its potential prognostic relevance.

## 5. Conclusions

This study demonstrated that a novel CTC detection method, combining high-resolution image scanning with negative enrichment, enables objective quantification of antigen expression and improves the CTC detection rate in patients with PDAC. These findings suggest that this method has potential as a reliable biomarker for the diagnosis of PDAC. Further large-scale, multicenter studies are warranted to validate its clinical utility and explore its application in patient stratification and treatment monitoring.

## 6. Patents

The authors declare that there are no patents resulting from the work reported in this manuscript.

## Figures and Tables

**Table 1:** Comparison of AUC for each threshold.

	Cutoff Value (Cells/5 mL)	AUC (95%CI)	SN	SP	PPV	NPV	Delong Test *p*-Value					
							EpCAM (99)	EpCAM (95)	EpCAM (90)	CSV (99)	CSV (95)	CSV (90)
EpCAM (99)	1	0.617 (0.414–0.785)	0.50	0.72	0.90	0.21	1.0000	0.0002	0.0063	0.3932	0.8864	0.6209
EpCAM (95)	2	0.830 (0.634–0.932)	0.74	0.76	0.96	0.33	0.0002	1.0000	0.2704	0.0002	0.0201	0.0021
EpCAM (90)	2	0.750 (0.456–0.914)	0.84	0.72	0.94	0.45	0.0063	0.2704	1.0000	0.0025	0.0551	0.0087
CSV (99)	1	0.570 (0.386–0.736)	0.39	0.72	0.88	0.18	0.3932	0.0002	0.0025	1.0000	0.5453	0.848
CSV (95)	1	0.605 (0.372–0.799)	0.63	0.57	0.85	0.17	0.8864	0.0201	0.0551	0.5453	1.0000	0.651
CSV (90)	2	0.581 (0.362–0.772)	0.50	0.72	0.89	0.19	0.6209	0.0021	0.0087	0.848	0.651	1.0000

EpCAM, epithelial cell adhesion molecule; CSV, cell surface vimentin; AUC, area under the curve; CI, confidence interval; SN, sensitivity; SP, specificity; PPV, positive predictive value; NPV, negative predictive value. ROC analyses were performed to evaluate the diagnostic performance of each threshold, and areas under the curve (AUC) were compared using the DeLong test. The optimal cutoff value was determined based on the maximum Youden index.

**Table 2:** Positivity of CTC and clinicopathological features in patients with PDAC.

		CTC ≥ 2	2 > CTC	*p*-Value
Age		67.1 (58–75)	72.9 (62–84)	0.079
Sex	Male	19	7	0.733
	Female	9	3	
Location	Head/Neck	11	7	0.092
	Body/Tail	18	3	
UICC Stage	I–II	17	7	0.598
	III–IV	11	3	
Tumor size	TS1-2	20	9	0.290
	TS3-4	8	1	
CY	0	21	8	0.747
	1	7	2	

CTC positivity was defined as detection of ≥2 cells; CTC, circulating tumor cells; PDAC, pancreatic ductal adenocarcinoma; CY, peritoneal cytology; UICC, Union for International Cancer Control. Statistical comparisons were performed using the Mann–Whitney U test for continuous variables and Fisher’s exact test for categorical variables.

## Data Availability

The datasets used and/or analyzed during the current study are not publicly available due to individual participants’ privacy but are available from the corresponding author on reasonable request.

## Supplementary Materials

The following supporting information can be downloaded at: https://www.mdpi.com/article/10.3390/cancers17223640/s1, Figure S1: Luminance was measured for both EpCAM and CSV; Table S1: Baseline characteristics of patients with PDAC; Table S2: Example of the Excel file used for ranking. This file contains a subset of representative images used to illustrate the machine-learning-based ranking process for cell and trash evaluations; Table S3: Number of candidate CTC at each threshold in patients with PDAC and controls; Method S1: Automated Cell Region Ranking Algorithm.

## Abbreviations

The following abbreviations are used in this manuscript:

PDAC	Pancreatic ductal adenocarcinoma
CTC	Circulating tumor cells
CSV	Cell surface vimentin
UICC	Union for International Cancer Control
DAPI	4′, 1, 6′-diamidino-2-phenylindole
ROC	Receiver Operating Characteristic
AUC	Areas under the curve
SN	Sensitivity
SP	Specificity
DL	Deep learning

## References

[1] Siegel R.L., Miller K.D., Jemal A. (2019). Cancer statistics, 2019. CA Cancer J. Clin..

[2] Igarashi T., Yamada S., Hoshino Y., Murotani K., Baba H., Takami H., Yoshioka I., Shibuya K., Kodera Y., Fujii T. (2023). Prognostic factors in conversion surgery following nab-paclitaxel with gemcitabine and subsequent chemoradiotherapy for unresectable locally advanced pancreatic cancer: Results of a dual-center study. Ann. Gastroenterol. Surg..

[3] Kanda M., Fujii T., Takami H., Suenaga M., Inokawa Y., Yamada S., Nakayama G., Sugimoto H., Koike M., Nomoto S. (2014). Combination of the serum carbohydrate antigen 19–9 and carcinoembryonic antigen is a simple and accurate predictor of mortality in pancreatic cancer patients. Surg. Today.

[4] Cabel L., Proudhon C., Gortais H., Loirat D., Coussy F., Pierga J.Y., Bidard F.C. (2017). Circulating tumor cells: Clinical validity and utility. Int. J. Clin. Oncol..

[5] Yeo D., Bastian A., Strauss H., Saxena P., Grimison P., Rasko J.E.J. (2022). Exploring the clinical utility of pancreatic cancer circulating tumor cells. Int. J. Mol. Sci..

[6] Allard W.J., Matera J., Miller M.C., Repollet M., Connelly M.C., Rao C., Tibbe A.G.J., Uhr J.W., Terstappen L.W.M.M. (2004). Tumor cells circulate in the peripheral blood of all major carcinomas but not in healthy subjects or patients with nonmalignant diseases. Clin. Cancer Res..

[7] Svensson C.M., Hübler R., Figge M.T. (2015). Automated classification of circulating tumor cells and the impact of Interobsever variability on classifier training and performance. J. Immunol. Res..

[8] Akashi T., Okumura T., Terabayashi K., Yoshino Y., Tanaka H., Yamazaki T., Numata Y., Fukuda T., Manabe T., Baba H. (2023). The use of an artificial intelligence algorithm for circulating tumor cell detection in patients with esophageal cancer. Oncol. Lett..

[9] Zeune L.L., Boink Y.E., van Dalum G., Nanou A., de Wit S., Andree K.C., Swennenhuis J.F., van Gils S.A., Terstappen L.W.M.M., Brune C. (2020). Deep learning of circulating tumour cells. Nat. Mach. Intell..

[10] Shen C., Rawal S., Brown R., Zhou H., Agarwal A., Watson M.A., Cote R.J., Yang C. (2023). Automatic detection of circulating tumor cells and cancer associated fibroblasts using deep learning. Sci. Rep..

[11] Brierley J., Gospodarowicz M.K., Wittekind C. (2025). TNM Classification of Malignant Tumor.

[12] Akita H., Nagano H., Takeda Y., Eguchi H., Wada H., Kobayashi S., Marubashi S., Tanemura M., Takahashi H., Ohigashi H. (2011). Ep-CAM is a significant prognostic factor in pancreatic cancer patients by suppressing cell activity. Oncogene.

[13] Mentink A., Isebia K.T., Kraan J., Terstappen L.W.M.M., Stevens M. (2023). Measuring antigen expression of cancer cell lines and circulating tumour cells. Sci. Rep..

[14] Nicolazzo C., Gradilone A., Loreni F., Raimondi C., Gazzaniga P. (2019). EpCAM^low^ circulating tumor cells: Gold in the waste. Dis. Markers.

[15] Rao C.G., Chianese D., Doyle G.V., Miller M.C., Russell T., Sanders R.A., Terstappen L.W.M.M. (2005). Expression of epithelial cell adhesion molecule in carcinoma cells present in blood and primary and metastatic tumors. Int. J. Oncol..

[16] Satelli A., Mitra A., Brownlee Z., Xia X., Bellister S., Overman M.J., Kopetz S., Ellis L.M., Meng Q.H., Li S. (2015). Epithelial-mesenchymal transitioned circulating tumor cells capture for detecting tumor progression. Clin. Cancer Res..

[17] Wei T., Zhang X., Zhang Q., Yang J., Chen Q., Wang J., Li X., Chen J., Ma T., Li G. (2019). Vimentin-positive circulating tumor cells as a biomarker for diagnosis and treatment monitoring in patients with pancreatic cancer. Cancer Lett..

[18] Gemenetzis G., Groot V.P., Yu J., Ding D., Teinor J.A., Javed A.A., Wood L.D., Burkhart R.A., Cameron J.L., Makary M.A. (2018). Circulating tumor cells dynamics in pancreatic adenocarcinoma correlate with disease status: Results of the prospective CLUSTER study. Ann. Surg..

[19] Poruk K.E., Valero V., Saunders T., Blackford A.L., Griffin J.F., Poling J., Hruban R.H., Anders R.A., Herman J., Zheng L. (2016). Circulating tumor cell phenotype predicts recurrence and survival in pancreatic adenocarcinoma. Ann. Surg..

[20] Munnings R., Gibbs P., Lee B. (2024). Evolution of Liquid Biopsies for Detecting Pancreatic Cancer. Cancers.

[21] Zhu P., Liu H.-Y., Liu F.-C., Gu F.-M., Yuan S.-X., Huang J., Pan Z.-Y., Wang W.-J. (2021). Circulating Tumor Cells Expressing Kruppel-Like Factor 8 and Vimentin as Predictors of Poor Prognosis in Pancreatic Cancer Patients. Cancer Control J. Moffitt Cancer Cent..

[22] Gasparini-Junior J.L., Fanelli M.F., Abdallah E.A., Chinen L.T.D. (2019). Evaluating Mmp-2 and Tgfs-Ri Expression in Circulating Tumor Cells of Pancreatic Cancer Patients and Their Correlation with Clinical Evolution. Arq. Bras. Cir. Dig. ABCD Braz. Arch. Dig. Surg..

[23] Cheng H., He W., Yang J., Ye Q., Cheng L., Pan Y., Mao L., Chu X., Lu C., Li G. (2020). Ligand-targeted polymerase chain reaction for the detection of folate receptor-positive circulating tumour cells as a potential diagnostic biomarker for pancreatic cancer. Cell Prolif..

[24] Chen J., Wang H., Zhou L., Liu Z., Tan X. (2022). A combination of circulating tumor cells and CA19-9 improves the diagnosis of pancreatic cancer. J. Clin. Lab. Anal..

[25] Ankeny J.S., Court C.M., Hou S., Li Q., Song M., Wu D., Chen J.F., Lee T., Lin M., Sho S. (2016). Circulating tumour cells as a biomarker for diagnosis and staging in pancreatic cancer. Br. J. Cancer.

[26] Fukasawa M., Watanabe T., Tanaka H., Itoh A., Kimura N., Shibuya K., Yoshioka I., Murotani K., Hirabayashi K., Fujii T. (2023). Efficacy of staging laparoscopy for resectable pancreatic cancer on imaging and the therapeutic effect of systemic chemotherapy for positive peritoneal cytology. J. Hepato-Bil. Pancreat. Sci..

[27] Court C.M., Ankeny J.S., Sho S., Winograd P., Hou S., Song M., Wainberg Z.A., Girgis M.D., Graeber T.G., Agopian V.G. (2018). Circulating tumor cells predict occult metastatic disease and prognosis in pancreatic cancer. Ann. Surg. Oncol..

[28] Bissolati M., Sandri M.T., Burtulo G., Zorzino L., Balzano G., Braga M. (2015). Portal vein-circulating tumor cells predict liver metastases in patients with resectable pancreatic cancer. Tumour Biol..

[29] Okubo K., Uenosono Y., Arigami T., Mataki Y., Matsushita D., Yanagita S., Kurahara H., Sakoda M., Kijima Y., Maemura K. (2017). Clinical impact of circulating tumor cells and therapy response in pancreatic cancer. Eur. J. Surg. Oncol..

[30] Martini V., Timme-Bronsert S., Fichtner-Feigl S., Hoeppner J., Kulemann B. (2019). Circulating tumor cells in pancreatic cancer: Current perspectives. Cancers.

