# Supplementary material for: Novel CTC Detection Method in Patients with Pancreatic Cancer Using High-Resolution Image Scanning
Source: Cancers (Basel). 2025 Nov 13;17(22):3640. doi: 10.3390/cancers17223640 (PMC12651565; doi:10.3390/cancers17223640)
Supplement: Supplementary file 1 [file cancers-17-03640-s001.zip › Supplementary_Method.pdf]

Automated Cell Region Ranking Algorithm

To assist semi-automated discrimination between cellular and non-cellular regions, a machine learning–based image-classification algorithm was employed. The algorithm was developed in collaboration with the Department of Mechanical and Intellectual Systems Engineering, University of Toyama (reference 8: Akashi T et al., *Oncol Lett* 26:320, 2023).Unlike the deep-learning approach used for independent cell detection in that study, the present algorithm was utilized solely for ranking purposes to support manual verification.

Machine-Learning–Based Ranking Model

Each individual image extracted from fluorescence microscopy data was characterized by quantitative features such as area, circularity, mean luminance, and channel intensity ratios. Two separate ranking procedures were applied: one to evaluate cells and another to evaluate trash. Each procedure calculated two numerical indices for every image: a kernel density estimation (KDE) score, representing the similarity to the feature distribution of reference samples, and a reconstruction error (Recon_error), representing the deviation between the input and reconstructed feature vectors. For both evaluations, images with higher KDE scores and lower reconstruction errors were considered more consistent with the target class (cell or trash). Images were automatically ranked according to these scores and listed in two separate Excel sheets corresponding to the "cell" and "trash" evaluations. All images, regardless of their rank, were manually reviewed and confirmed by two trained evaluators before inclusion in subsequent analyses.

Training Dataset

The algorithm was trained on an annotation library containing approximately 10,000 fluorescence image patches, including both cellular and non-cellular regions, collected under identical imaging conditions using the BZ-X800 fluorescence microscope (KEYENCE, Osaka, Japan). These images were manually annotated and curated by trained reviewers prior to model training. No separate test dataset was prepared, as the model was designed exclusively for ranking assistance rather than for diagnostic classification.

Role in the Analysis

The algorithm was designed as an auxiliary pre-screening tool to facilitate annotation efficiency and consistency. It did not perform automatic binary classification, nor was it used for diagnostic or statistical decisions such as threshold determination or CTC positivity.

All downstream quantitative analyses (e.g., luminance measurement and threshold optimization) were conducted using human-verified cell images only.

Reference Performance

Independent performance evaluation (accuracy, sensitivity, specificity) was not conducted in this study, as the algorithm served exclusively for ranking assistance. For technical reference, the base CNN model reported by Akashi et al. achieved >99% accuracy in distinguishing cancer cells from peripheral blood mononuclear cells under similar imaging conditions (reference 8: Akashi T et al., *Oncol Lett* 26:320, 2023) .
