# Supplementary figures and images for: Novel CTC Detection Method in Patients with Pancreatic Cancer Using High-Resolution Image Scanning
Source: Cancers (Basel). 2025 Nov 13;17(22):3640. doi: 10.3390/cancers17223640 (PMC12651565; doi:10.3390/cancers17223640)

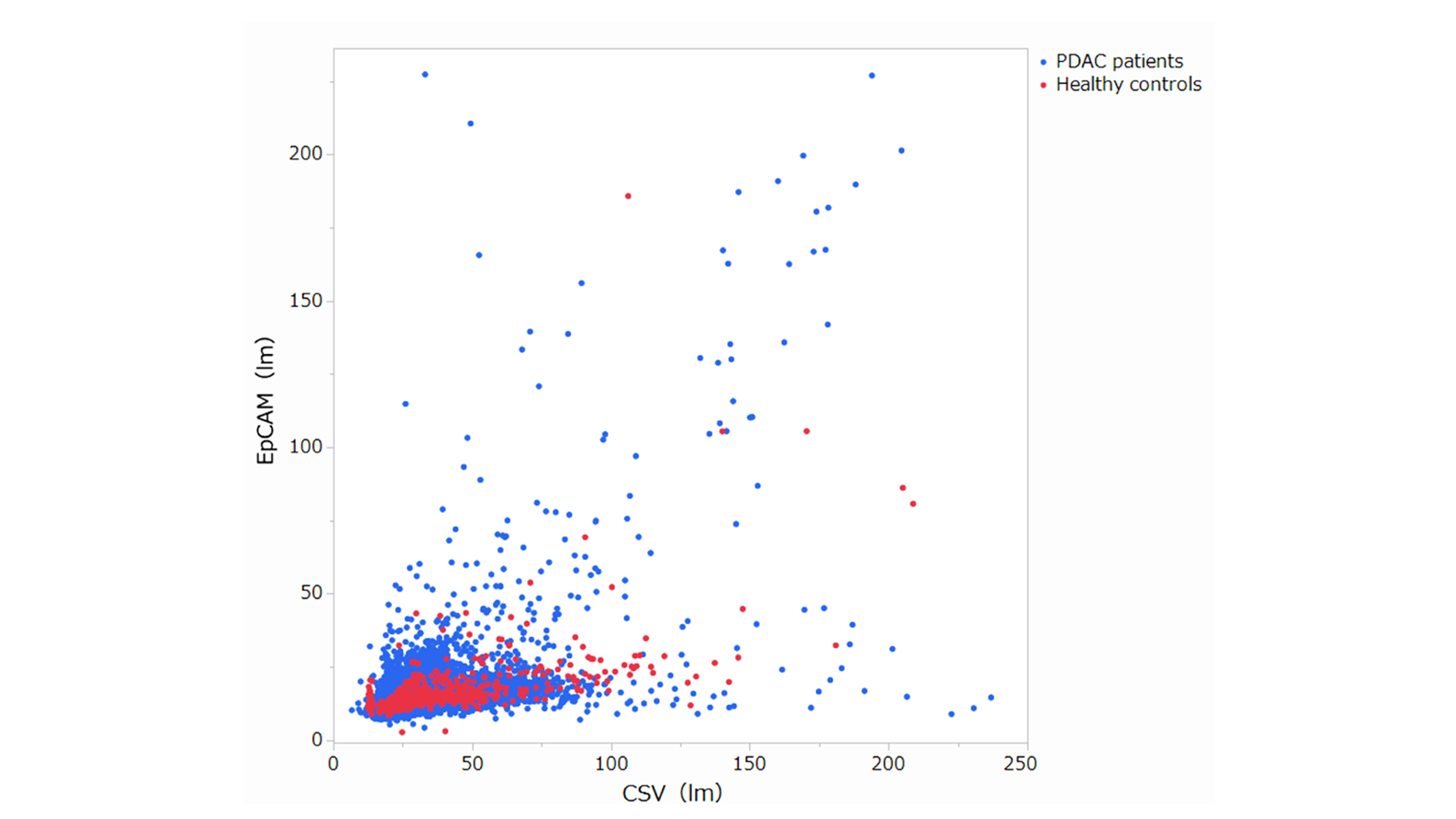

Supplement: Supplementary file 1 [file cancers-17-03640-s001.zip › Supplymentary_Figure1.tif]
